# The ecological significance of birds feeding from the hand of humans

**DOI:** 10.1038/s41598-020-66165-9

**Published:** 2020-06-17

**Authors:** Anders Pape Møller, Canwei Xia

**Affiliations:** 1 Ministry of Education Key Laboratory for Biodiversity Science and Ecological Engineering, College of Life Sciences, Beijing Normal University, Beijing, 100875 China; 2 Ecologie Systématique Evolution, Université Paris-Sud, CNRS, AgroParisTech, Université Paris-Saclay, F-91405 Orsay, Cedex France

**Keywords:** Behavioural ecology, Evolutionary ecology, Zoology

## Abstract

Animals keep a safe distance to humans and thus humans rarely physically encounter wild animals. However, birds have been known to feed from the hand of humans. Such behaviour must reflect the trade-off between acquisition of food and the risk of being captured by a potential predator feeding from the hand. Relying on YouTube, an international video-sharing platform, we found 36 European bird species recorded feeding from the hand of humans. We compared ecological traits between these species and all other 490 European bird species, which were not recorded as feeding from a human hand. We found that species with a large number of innovative behaviours, a higher rate of introduction success, larger breeding range, larger population size, and urban tolerance have a higher probability of feeding from the hand of a human. These associations were also supported after control for the similarity among taxa due to common phylogenetic descent. In conclusion, these findings suggest that frequent feeding from the hand of a human results in the transition from natural environments to novel urbanized environments with consequences for population size increasing and range expansion.

## Introduction

Animals are usually afraid of humans from whom they keep a safe distance. Therefore, it is surprising that there are numerous photos of humans feeding birds from the hand on YouTube and other web sites. For wild animals who feed from the hand of a human, this is a potentially lethal activity if captured. Any such behaviour may hence be costly in terms of reduced viability. If humans commonly provide particularly valuable morsels when feeding a wild animal, there should be a trade-off between acquisition of food and the mortality costs of feeding from the hand of a human for a wild animal. Indeed, animals are known to keep a safe distance of sometimes as much as several hundred meters to humans as reflected by the flight initiation distance, the distance when an animal takes flight when approached by a human being^1–4^. Such movements associated with flight initiation distance may occur numerous times per day in urban environments where human population density is high and humans thus particularly often disturb animals^2,5^.

Populations of wild animals are generally food limited^6^, and thus they may run considerable risks when acquiring even small amounts of food. Feeding events when animals acquire food from the hand of a human have been described numerous times ranging from humans attracting house sparrows *Passer domesticus* in Hyde Park in London, UK, to Tibetan monks feeding white-eared pheasants *Crossoptilon crossoptilon*, humans in remote forests during winter feeding gray jays *Perisoreus canadensis* from the hand, and to a human training an adult wild white-tailed sea eagle *Haliaeetus albicilla* to eat from the hand. Although feeding from the hand by humans has been reported frequently on the internet, there is no review of this behaviour and its association with ecological traits. Here we provide such a review by attempting to identify the ecological significance of this behaviour.

The objectives of this study were to test a number of associations derived from animal responses to human attempts to feed birds from the hand. We tested (1) whether the occurrence of feeding from the hand of a human was positively related to the number of innovative behaviours as reflected by novel and unusual behaviour reported in the literature^7^. (2) As close contact between human and wild birds is more common in urban than in rural habitats^8^, we should expect a positive association between feeding of food from the hand and urban tolerance in birds. (3) Species feeding from the hand of humans may benefit to expansion to novel environments. Therefore, we should expect a positive correlation between introduction success to novel environments and consumption of food from the hand^9^. (4) Bird species that are accustomed to the presence of humans should also be those that most readily learn to eat food from the hand. Thus, we would expect a negative correlation between flight initiation distance and the probability of food consumption from the hand of a human. (5) If some individuals learn to feed from the hand of a human, such individuals should survive and reproduce faster, resulting in a more rapid spread across small and subsequently large spatial scales. Hence, we should expect individuals of such species to expand their geographical range increasing in population size because of a larger range, but also because of an increase in survivorship. In this study, we tested the above predictions, using extensive data from YouTube (www.youtube.com).

## Results

Totally, 36 species were recorded feeding from the hand of humans. Among these 36 species, 23 species belong to Passeriformes, including 6 finches (*Carduelis chloris*, *Carduelis hornemanni*, *Carduelis flammea*, *Pinicola enucleator*, *Loxia curvirostra*, *Loxia leucoptera*), 5 tits (*Parus palustris*, *Parus montanus*, *Parus ater*, *Parus major*, *Parus caeruleus*), 2 chats (*Monticola solitarius*, *Erithacus rubecula*), 2 crows (*Perisoreus infaustus*, *Corvus corax*), 2 sparrows (*Passer domesticus*, *Passer montanus*), 1 starling (*Sturnus vulgaris*), 1 thrushe (*Turdus merula*), 1 nuthatch (*Sitta europaea*), 1 swallows (*Hirundo rustica*), 1 waxwing (*Bombycilla garrulus*), 1 bunting (*Emberiza citrinella*); 13 species are non-Passeriformes, including 3 Anseriformes (*Cygnus olor*, *Anser anser*, *Branta leucopsis*), 2 Charadriiformes (*Larus ridibundus*, *Rissa tridactyla*), 2 Gruiformes (*Gallinula chloropus*, *Fulica atra*), 2 Columbiformes (*Columba livia*, *Streptopelia decaocto*), 1 Ciconiiformes (*Ciconia ciconia*), 1 Pelecaniformes (*Ardea cinerea*), 1 Suliformes (*Phalacrocorax carbo*), 1 Accipitriformes (*Haliaeetus albicilla*).

Information of at least one ecological variable (Number of Innovations, Introduction Success, Flight Initiation Distance, Breeding Range, Population Size, Urban Tolerance) was collected for these 36 species, and also for all 490 other European bird species which were not recorded as feeding from a human hand. Based on these data, species with a large Number of Innovative Behaviours, a higher rate of Introduction Success, larger Breeding Range, larger Population Size, and Urban Tolerance have a higher probability of feeding from the hand of a human (Table 1, Supplementary Fig. 1).

**Table 1:** Comparing ecological traits, and assessing the relationship between ecological traits and species feeding from the hand of a human based on Logistic Regression Models.

Ecological variables	Species feeding from hand +	Species not feeding from hand +	Coefficient	Standard error	z	P
Number of innovations*	9.45 ± 1.48 (29)	4.62 ± 0.33 (193)	2.57	0.61	4.21	<0.001
Introduction success (%)	0.58 ± 0.10 (10)	0.19 ± 0.06 (27)	3.21	1.21	2.66	0.008
Flight initiation distance (m)*	16.49 ± 4.64 (23)	17.86 ± 1.34 (110)	−1.49	0.79	-1.90	0.058
Breeding range (million kilometre^^2^)*	22.14 ± 2.81 (23)	15.11 ± 0.52 (110)	10.13	3.46	2.93	0.003
Population size (million)*	24.3 ± 5.44 (23)	10.10 ± 1.98 (110)	0.96	0.35	2.70	0.007
Urban tolerance	16 / 36	48 / 490	2.00	0.37	5.42	<0.001

^*^These variables were log_10_-transformed in the model.

+ Data were reported as mean ± standard error (sample size), except “Urban tolerance” (number of urban species / total number of species).

The differences between species feeding and not feeding from a human hand were also significant after control for similarity among taxa due to common phylogenetic descent. In Phylogenetic Regression Models, species feeding from a human hand are significantly related to a larger Number of Innovative behaviours, a higher rate of Introduction Success, larger Breeding Range, larger Population Size, and higher probability of Urban Tolerance (Table 2). Flight Initiation Distance is shorter in species feeding from a human hand, but the difference is not significant either controlling or not controlling for the statistical dependence of a shared evolutionary ancestry (Tables 1, 2).

**Table 2:** Relationships between ecological traits and species feeding from a human hand based on Phylogenetic Regression Models.

Ecological variables	Coefficient	Standard error	z	Sample size	P	Phylogenetic signal +
Number of innovations*	2.18	0.55	3.98	230	<0.001	1.97
Introduction success (%)	3.24	1.23	2.64	37	0.008	0.46
Flight initiation distance (m)*	−1.79	1.04	−1.72	133	0.086	1.82
Breeding range (million kilometre^2)*	10.84	3.86	2.81	133	0.005	1.77
Population size (million)*	1.22	0.46	2.65	133	0.008	2.11
Urban tolerance	1.94	0.41	4.78	526	<0.001	1.68

*These variables were log_10_-transformed in the model.

+Phylogenetic signal was measured as the scalar magnitude of the phylogenetic variance-covariance matrix.

## Discussion

Although feeding from the hand by humans has been reported frequently on the internet, little is known about the ecological significance of this behaviour. This study assesses the association between this behaviour and ecological traits, and found that the consumption of food from the hands of humans has several perspectives. First, the study suggests a role for cognition in human-animal interactions because species that are likely to consume food from the hand of a human also have a high number of innovation behaviours. Second, consumption of food from the hand of humans might imply that macro-ecology accounts for some of the patterns. The link between birds feeding from the hand of a human and population size and range size suggests that bird species feeding from the hand of humans may result in expansion of range size and population size. Furthermore, these birds may benefit to expansion to novel environments, with a higher rate of introduction success and higher probability of urban tolerance. Third, we hypothesized that avian consumption of food from the hand of a human may be accustomed to the presence of humans, i.e. decreasing flight initiation distance. This hypothesis was partly supported in the study. Flight initiation distance is shorter in species feeding from a human hand, but the difference is not significant.

There are two potential biases which may affect the interpretation of this study. First, Type I error rate may be inflated in the analysis, as multiple statistical comparisons on the same data set were used to assess the associations with six ecological traits. However, the P values in the analysis are either larger than 0.05 or lower than 0.008. Thus, the conclusion did not change even following Bonferroni correction^10^ or when the Holm–Bonferroni method^11^ was used to adjust the alpha level in the statistics. Second, data from citizen science projects always violate the assumption that observations are unbiased^12–14^. In this study, we scored whether bird species are feeding from a human hand based on photos and videos in web searches, which may bias the observation towards colourful, urban, and common species^15^. In that way, the association between feeding from a hand and ecological traits may simply be due to more observations of these species. However, we did not fully understand people’s proclivity to upload images and video, so this potential bias is difficult to control.

In conclusion, we provide a review of bird species feeding from a human hand, and find that it is associated with other ecological traits (number of innovation behaviours, introduction success, breeding range, population size, urban tolerance). These associations are robust to the control for similarity among taxa due to common phylogenetic descent, and adjusting the alpha level to avoid an inflated Type I error rate in statistics. As birds feeding from a human hand reflect human-animal interactions and associate with other ecological traits, this behaviour should get more attention in further research.

## Methods

### Data collection

An extensive search was made on the internet for photos and videos of birds feeding from the hand of a human. Such information was searched using YouTube, an international video-sharing platform. The keywords feed*, food, hand, human, proximity, flight initiation distance, flight were used in searches. Whenever a new photo or video was detected, all entries for persons were also searched in order to locate additional information. These persons were asked for additional information if necessary, e.g. the location of the photo or video; whether the bird is wild or artificial raised. Only wild European bird species were included in this study to avoid heterogeneity in data among continents. Whether each species feeding from the hand was scored as Yes or No, based on whether at least one individual of this species was recorded feeding from the hand.

A current extended version of the database on avian innovations collected by Overington and colleagues was used in this study^7^. The database contains 2182 innovation reports for 803 species in 76 families, compiled from volumes of 64 ornithology journals published between 1944 and 2002. Reports were included in the database if they contained keywords such as “novel”, “opportunistic”, “first description”, “not noted before” or “unusual”. For a detailed description of the systematic data collection, see Overington *et al*.^7^. The Number of Innovations is known to correlate positively with a large number of ecological variables^16^, such as tool use and learning^17^, successful introduction of birds to non-native locations^18^, and higher richness of subspecies^19^.

Urbanization is the process that allows animals to invade, become established, expand and successfully multiply when entering urban habitats^8,20–22^. Previous studies have shown that indices of urbanization of birds in cities such as the estimated first year when urbanized, whether a species is urbanized or not, whether it breeds in urban centres or not, and the difference in population density between urban and nearby rural habitats are all strongly positively correlated^23^. Whether a species was urban tolerant was scored (yes/no), using the habitat information from Burfield and van Bommel^24^, following the criterion in Hu and Cardoso^25^, e.g. a species was classified as urban tolerant when its habitat description mentioning any human-built structures or severely human-altered environments such as towns, streets, industrial buildings and airports.

Studies of introduction success to oceanic islands provide crucial information on the ability of different species to successfully become established in novel habitats^26^. Introduction success was the number of oceanic islands that were successfully colonized divided by the total number of oceanic islands in which the birds were released^26–28^. The main factor determining introduction success is introduction effort measured as the number of individuals that are released^27,28^. Urbanized birds are more likely to become successfully established than rural species^8,9^. The data on Introduction Success rate were from Møller *et al*.^9^.

Flight initiation distance is recorded as the nearest distance between an individual bird and the closest proximity of a human being, and as such it provides a measure of the risk that an individual animal takes when approached by a human^1,3,4^. Previous studies of flight initiation distance have shown significant repeatability of this measurement among species, observers, sites, years, and countries^29,30^. These data on Flight Initiation Distance were from Møller and Garamszegi^29^.

Information on breeding population size and breeding range in the Western Palaearctic was used in this study^24^. These data (Population Size and Breeding Range) were from Møller and Garamszegi^29^.

### Statistical analyses

Four variables (Number of Innovations, Flight Initiation Distance, Breeding Range, Population Size) were log_10_-transformed to meet approximately normal distribution. Logistic regression models were employed to assess the relationship between ecological traits and species feeding from the hand of a human. Six models were built. Feeding from a Hand was the response variable with a binomial error distribution, while Number of Innovations, Introduction Success, Flight Initiation Distance, Breeding Range, Population Size and Urban Tolerance was the predictor variable, respectively, in each model.

To account for statistical dependence of observations due to shared evolutionary ancestry, a Phylogenetic Regression Model for Binary Dependent Variable was built. 1000 phylogenetic trees were retrieved from birdtree.org^31^, which were merged into a maximum clade credibility tree using Tree Annotator in BEAST v1.8.3^32^. Six models were built, with Feeding from a Hand as a response variable and Number of Innovations, Introduction Success, Flight Initiation Distance, Breeding Range, Population Size and Urban Tolerance as a predictor variable, respectively, in each model.

We did not include all predictor variables (Number of Innovation, Introduction Success, Flight Initiation Distance, Breeding Range, Population Size, Urban Tolerance) in one model, as only 26 species had information for all these variables. We could not find any robust association with such a small sample size. The data used in this study can be seen in Supplementary Table S1.

All analyses were conducted using R 3.5.0 (R Core Team 2018). Phylogenetic Regression Models were performed using the “ape” package^33^. Data were reported as mean ± standard error. Results were considered significant if P < 0.05 (two-tailed test).

### Ethical approval

All applicable international, national, and/or institutional guidelines for the care and use of animals were followed. This article does not contain any studies with animals performed by any of the authors.

## Supplementary information

Supplementary Information.

## Data Availability

All the data used in this study can be seen in Supplementary Table S1.
