# Supplementary material for: The ecological significance of birds feeding from the hand of humans
Source: Sci Rep. 2020 Jun 17;10:9773. doi: 10.1038/s41598-020-66165-9 (PMC7300022; doi:10.1038/s41598-020-66165-9)
Supplement: Supplementary file 1 — Supplementary Information. [file 41598_2020_66165_MOESM1_ESM.pdf]

The ecological significance of birds feeding from the hand of humans

Anders Pape Møller[1,2] and Canwei Xia[1]

[1] Ministry of Education Key Laboratory for Biodiversity Science and Ecological Engineering, College of Life Sciences, Beijing Normal University, Beijing 100875, China

[2] Ecologie Systématique Evolution, Université Paris-Sud, CNRS, AgroParisTech, Université Paris-Saclay, F-91405 Orsay Cedex, France

Email addresses: anders.moller@u-psud.fr, xiacanwei@bnu.edu.cn

Correspondence to APM:

Tel: (+33) 1 69 15 56 88

Fax: (+33) 1 69 15 56 96

Running headline: A. P. Møller and C. Xia: Ecology of birds feeding from your hand

Supplementary Figure 1. Comparing ecological traits (a. Number of Innovations, b. Introduction Success, c. Flight Initiation Distance, d. Breeding Range, e. Population Size) between species feeding and not feeding from a human hand.

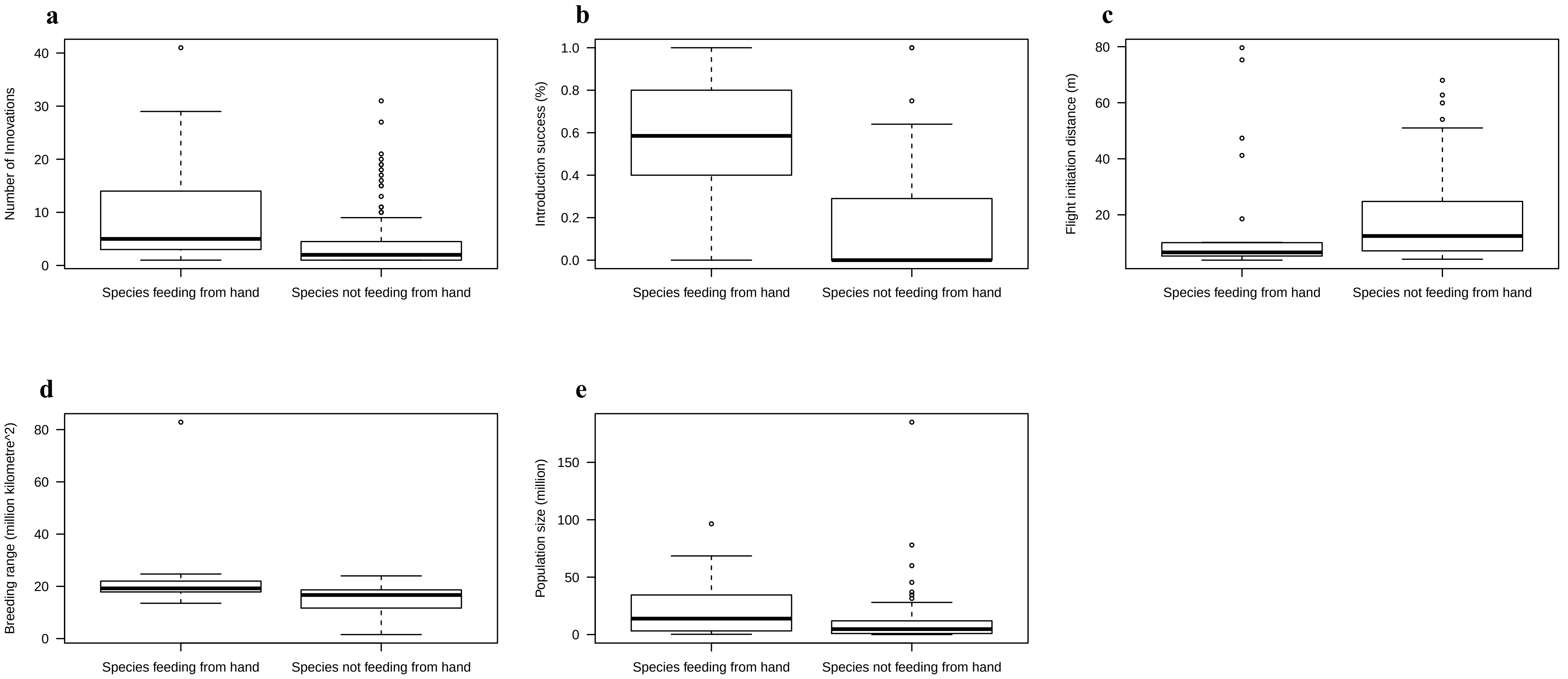

a
Number of Innovations
0
10
20
30
40
Species feeding from hand
Species not feeding from hand
b
Introduction success (%)
0.0
0.2
0.4
0.6
0.8
1.0
Species feeding from hand
Species not feeding from hand
c
Flight initiation distance (m)
20
40
60
80
Species feeding from hand
Species not feeding from hand
d
Breeding range (million kilometre^2)
0
20
40
60
80
Species feeding from hand
Species not feeding from hand
e
Population size (million)
0
50
100
150
Species feeding from hand
Species not feeding from hand

Supplementary Table S1. Information on whether bird species were scored as feeding from human hand, and six ecological traits (Number of Innovation behaviours, Introduction Success rate, Flight Initiation Distance, Breeding Range, Population Size, Urban Tolerance).

Species	Feeding from hand	Number of innovations	Urban tolerance	Introduction success (%)	Flight initiation distance (m)	Breeding range (million km2)	Population size (thousand)
Accipiter brevipes	No		No				
Accipiter gentilis	No	17	No				
Accipiter nisus	No	7	No		10	20.21	395
Acrocephalus agricola	No		No				
Acrocephalus arundinaceus	No		No				
Acrocephalus brevipennis	No		No				
Acrocephalus dumetorum	No	1	No				
Acrocephalus melanopogon	No		No				
Acrocephalus paludicola	No		No				
Acrocephalus palustris	No		No		8.84	12.99	5000

Acrocephalus schoenobaenus	No		No		7.56	16.95	5900
Acrocephalus scirpaceus	No		No		6.73	16.34	3850
Acrocephalus stentoreus	No		No				
Actitis hypoleucos	No		No		17	18.57	1160
Aegithalos caudatus	No	5	Yes		5.02	18.28	8500
Aegolius funereus	No		No				
Aegypius monachus	No		No				
Alaemon alaudipes	No		No				
Alauda arvensis	No	3	No	0.64	31.37	18.63	60000
Alca torda	No		No				
Alcedo atthis	No	3	No		16.27	14.92	120
Alectoris barbara	No		No				
Alectoris chukar	No		No				
Alectoris graeca	No	1	No				
Alectoris rufa	No	2	No	0.09	35.54	2.54	3250
Alle alle	No		No				
Ammomanes cinctura	No		No				
Ammomanes deserti	No		No				
Anas acuta	No		No				
Anas clypeata	No		No				
Anas crecca	No		No		39.23	21.26	1060
Anas penelope	No	3	No				

Anas platyrhynchos	No	10	Yes	0.33	13.42	22.39	4200
Anas querquedula	No		No				
Anas strepera	No	1	No				
Anser albifrons	No	3	No				
Anser anser	Yes	3	Yes				
Anser brachyrhynchus	No		No				
Anser erythropus	No	1	No				
Anser fabalis	No	1	No				
Anthreptes metallicus	No		No				
Anthreptes platurus	No		No				
Anthus berthelotii	No		No				
Anthus campestris	No		No				
Anthus cervinus	No		No				
Anthus gustavi	No		No				
Anthus hodgsoni	No		No				
Anthus novaeseelandiae	No		No				
Anthus pratensis	No		No		13.97	17.60	11500
Anthus spinoletta	No	2	No		4.16	19.60	1520
Anthus trivialis	No		No		10.19	18.11	34500
Apus affinis	No		Yes				
Apus apus	No	1	Yes		38.1	18.29	11950
Apus caffer	No		Yes				
Apus pallidus	No		Yes				

Apus unicolor	No	1	Yes				
Aquila adalberti	No		No				
Aquila chrysaetos	No	11	No				
Aquila clanga	No		No				
Aquila fasciatus	No		No				
Aquila heliaca	No		No				
Aquila nipalensis	No		No				
Aquila pomarina	No	1	No				
Ardea cinerea	Yes	28	Yes		47.36	19.72	250
Ardea purpurea	No	3	No				
Ardeola ralloides	No	2	No				
Arenaria interpres	No	11	No		20.5	3.18	58
Asio flammeus	No		No				
Asio otus	No		No				
Athene noctua	No		No	1	36.43	14.33	930
Aythya ferina	No		No				
Aythya fuligula	No	1	No		10.68	18.58	805
Aythya marila	No	2	No				
Aythya nyroca	No		No				
Bombycilla garrulus	Yes	4	No				
Bonasa bonasia	No		No				
Botaurus stellaris	No	1	No				
Branta bernicla	No	4	No		23.5	4.41	2
Branta leucopsis	Yes		No				
Bubo bubo	No		No				

Bubo scandiaca	No		No				
Bubulcus ibis	No	16	No		24.38	8.95	102
Bucanetes githagineus	No		No		5.01	5.16	16
Bucephala clangula	No	1	No				
Bucephala islandica	No		No				
Bulweria bulwerii	No		No				
Burhinus oedicnemus	No	1	No				
Buteo buteo	No	21	No		54.06	20.99	955
Buteo lagopus	No	2	No				
Buteo rufinus	No		No				
Calandrella brachydactyla	No		No				
Calandrella rufescens	No		No				
Calcarius lapponicus	No		No				
Calidris alba	No	5	No		18	6.17	38
Calidris alpina	No	2	No				
Calidris canutus	No	2	No				
Calidris maritima	No	2	No				
Calidris minuta	No	1	No				
Calidris temminckii	No		No				
Calonectris diomedea	No	1	No				
Caprimulgus europaeus	No	1	No				
Caprimulgus	No		No				

ruficollis							
Carduelis cannabina	No	2	Yes	0	9.25	18.74	19000
Carduelis carduelis	No	4	Yes	0.25	7.63	19.35	20500
Carduelis chloris	Yes	8	Yes	0.5	6.59	22.12	23000
Carduelis flammea	Yes	5	Yes	1	4.5	13.53	13900
Carduelis flavirostris	No	1	No				
Carduelis hornemanni	Yes		No				
Carduelis spinus	No	5	No	0	4.83	18.13	14000
Carpodacus erythrinus	No		No				
Carpodacus rubicilla	No		No				
Carpodacus synoicus	No		No				
Carpospiza brachydactyla	No		No				
Casmerodius albus	No	11	No				
Catharacta skua	No	6	No				
Cepphus grylle	No		No				
Cercomela melanura	No		No				
Certhia brachydactyla	No		No		7.08	9.57	6200
Certhia familiaris	No	2	No				
Cettia cetti	No		No		5.55	10.98	1100
Charadrius alexandrinus	No	1	No				

Charadrius dubius	No	1	No				
Charadrius hiaticula	No	1	No		19.65	15.18	170
Chersophilus duponti	No		No				
Chlamydotis undulata	No		No				
Chlidonias hybrida	No		No				
Chlidonias leucopterus	No	4	No				
Chlidonias niger	No	5	No				
Ciconia ciconia	Yes	6	Yes				
Ciconia nigra	No	1	No				
Cinclus cinclus	No	4	No				
Circaetus gallicus	No		No				
Circus aeruginosus	No		No				
Circus cyaneus	No	9	No				
Circus macrourus	No	1	No				
Circus pygargus	No	5	No				
Cisticola juncidis	No		No		5.32	11.18	665
Clamator glandarius	No		No				
Clangula hyemalis	No	2	No				
Coccothraustes coccothraustes	No	3	Yes				
Columba bollii	No		No				
Columba junoniae	No		No				
Columba livia	Yes	1	Yes		6.46	22.01	12150

Columba oenas	No		No				
Columba palumbus	No	2	Yes	0	19.69	20.99	13000
Columba trocaz	No		No				
Coracias garrulus	No	3	No				
Corvus corax	Yes	17	No		79.63	24.72	710
Corvus cornix	No		Yes				
Corvus corone	No	31	Yes		27.48	18.65	12000
Corvus frugilegus	No	9	Yes	0.75	44.39	16.76	14000
Corvus monedula	No	4	Yes	0	26.67	17.13	10100
Corvus ruficollis	No		No				
Coturnix coturnix	No		No	0			
Crex crex	No		No				
Cuculus canorus	No	1	No		25.89	18.42	6400
Cuculus saturatus	No		No				
Cursorius cursor	No		No				
Cyanopica cyanus	No		No	0			
Cygnus columbianus	No		No				
Cygnus cygnus	No		No				
Cygnus olor	Yes	1	No	0.4			
Delichon urbicum	No		Yes		6.34	18.41	16950
Dendrocopos leucotos	No	1	No				
Dendrocopos major	No	9	Yes		13.44	19.51	15000
Dendrocopos medius	No		No				
Dendrocopos minor	No	2	No				

Dendrocopos syriacus	No	1	No				
Dryocopus martius	No		No		38.71	17.13	1070
Egretta garzetta	No	13	No		24.5	10.94	81
Elanus caeruleus	No		No				
Emberiza aureola	No		No				
Emberiza bruniceps	No		No				
Emberiza buchanani	No		No				
Emberiza caesia	No		No				
Emberiza cia	No		No		9.43	10.61	2700
Emberiza cineracea	No		No				
Emberiza cirlus	No		No				
Emberiza citrinella	Yes		No	0.25	9.99	15.71	24500
Emberiza hortulana	No	1	No	0			
Emberiza leucocephalos	No		No				
Emberiza melanocephala	No		No				
Emberiza pallasi	No		No				
Emberiza pusilla	No		No				
Emberiza rustica	No		No				
Emberiza schoeniclus	No	2	No	0	9.81	18.65	6800
Emberiza striolata	No		No				
Eremalauda dunni	No		No				
Eremophila alpestris	No		No				

Eremophila bilopha	No		No				
Eremopsaltria mongolicus	No		No				
Eremopterix nigriceps	No		No				
Erithacus rubecula	Yes	6	Yes	0	5.13	22.01	63000
Erythropygia galactotes	No		No				
Eudromias morinellus	No	1	No				
Falco biarmicus	No	2	No				
Falco cherrug	No		No				
Falco columbarius	No	10	No				
Falco eleonorae	No	1	No				
Falco naumanni	No		Yes				
Falco peregrinus	No	19	Yes				
Falco rusticolus	No		No				
Falco subbuteo	No	6	No				
Falco tinnunculus	No		Yes		30.94	22.23	415
Falco vespertinus	No	1	No				
Ficedula albicollis	No	1	No				
Ficedula hypoleuca	No	3	Yes				
Ficedula parva	No	1	No				
Ficedula semitorquata	No		No				

Francolinus francolinus	No		No				
Fratercula arctica	No		No				
Fringilla coelebs	No	7	Yes	0.17	7.14	22.45	185000
Fringilla montifringilla	No	2	No	0			
Fringilla teydea	No		No				
Fulica atra	Yes	3	No		18.57	20.44	1800
Fulica cristata	No		No				
Fulmarus glacialis	No	1	No				
Galerida cristata	No		Yes		14.33	14.67	5600
Galerida theklae	No		No		7.18	5.08	1800
Gallinago gallinago	No	1	No		25.83	22.45	1415
Gallinago media	No		No				
Gallinago stenura	No		No				
Gallinula chloropus	Yes	12	Yes		9.88	82.85	1300
Garrulus glandarius	No	6	Yes		11.76	17.96	9500
Gavia arctica	No	1	No				
Gavia immer	No	2	No				
Gavia stellata	No	1	No				
Geronticus eremita	No		No				
Glareola nordmanni	No		No				
Glareola pratincola	No	1	No				
Glaucidium passerinum	No		No				

Grus grus	No		No				
Grus virgo	No		No				
Gypaetus barbatus	No	1	No				
Gyps fulvus	No	1	No				
Haematopus ostralegus	No	2	Yes				
Haliaeetus albicilla	Yes		No				
Hieraaetus pennatus	No		No				
Himantopus himantopus	No	1	No				
Hippolais caligata	No		No				
Hippolais icterina	No		Yes		7.52	14.13	5300
Hippolais languida	No		No				
Hippolais olivetorum	No		No				
Hippolais pallida	No		No				
Hippolais polyglotta	No	1	No				
Hirundo daurica	No	1	No				
Hirundo rupestris	No	1	No				
Hirundo rustica	Yes	18	Yes		10.16	18.42	26000
Histrionicus histrionicus	No		No				
Hydrobates pelagicus	No	1	No				
Hypocolius ampelinus	No		No				
Irania gutturalis	No		No				

Ixobrychus minutus	No		No				
Jynx torquilla	No	1	No				
Lagopus lagopus	No		No				
Lagopus muta	No		No				
Lanius collurio	No	4	No		7.27	9.35	9650
Lanius excubitor	No	18	No		21.16	20.10	325
Lanius isabellinus	No		No				
Lanius minor	No	3	No				
Lanius nubicus	No		No				
Lanius senator	No	2	No		10.99	11.69	840
Larus argentatus	No	27	Yes		40.15	12.40	1525
Larus armenicus	No		No				
Larus audouinii	No		No				
Larus canus	No	8	No		59.94	12.30	1045
Larus fuscus	No	2	No		37	18.67	325
Larus genei	No		No				
Larus glaucoides	No	1	No				
Larus hyperboreus	No	1	No				
Larus ichthyaetus	No		No				
Larus marinus	No	8	No		68	24.01	145
Larus melanocephalus	No	2	No				
Larus minutus	No	2	No				
Larus ridibundus	Yes	21	No		41.2	19.69	1850
Limicola falcinellus	No		No				

Limosa lapponica	No		No				
Limosa limosa	No		No				
Locustella fluviatilis	No		No				
Locustella lanceolata	No		No				
Locustella luscinioides	No		No				
Locustella naevia	No		No		15.03	15.49	1520
Loxia curvirostra	Yes	5	No		4.74	17.61	9400
Loxia leucoptera	Yes	2	No				
Loxia pytyopsittacus	No		No				
Loxia scotica	No		No				
Lullula arborea	No		No	0	12.14	14.54	2300
Luscinia calliope	No		No				
Luscinia luscinia	No		No		15.89	10.19	5300
Luscinia megarhynchos	No		No	0	8.16	11.84	8100
Luscinia svecica	No		No				
Lymnocryptes minimus	No		No				
Marmaronetta angustirostris	No		No				
Melanitta fusca	No	1	No				
Melanitta nigra	No		No				
Melanocorypha bimaculata	No		No				

Melanocorypha calandra	No		No				
Melanocorypha leucoptera	No		No				
Melanocorypha yeltoniensis	No		No				
Mergellus albellus	No		No				
Mergus merganser	No	1	No				
Mergus serrator	No	1	No				
Merops apiaster	No	4	No		37.02	13.58	740
Miliaria calandra	No		No		9.13	15.79	14950
Milvus migrans	No	15	No		37.91	20.34	82
Milvus milvus	No	2	No				
Monticola saxatilis	No		No		16.25	13.41	210
Monticola solitarius	Yes	1	No				
Montifringilla nivalis	No		No				
Morus bassanus	No	2	No				
Motacilla alba	No	9	Yes		11.32	22.42	19500
Motacilla cinerea	No	1	No		7.07	22.13	1170
Motacilla citreola	No		No				
Motacilla flava	No	2	No		11.24	20.15	10950
Muscicapa striata	No	6	Yes		8.5	18.45	18000
Nectarinia osea	No		Yes				
Neophron percnopterus	No	5	No				

Netta rufina	No	1	No				
Nucifraga caryocatactes	No	1	No				
Numenius arquata	No	3	No		62.75	13.18	290
Numenius phaeopus	No		No				
Nycticorax nycticorax	No	2	No				
Oceanodroma castro	No		No				
Oceanodroma leucorhoa	No	1	No				
Oenanthe albonigra	No		No				
Oenanthe deserti	No		No				
Oenanthe finschii	No		No				
Oenanthe hispanica	No		No				
Oenanthe isabellina	No		No				
Oenanthe leucopyga	No		No				
Oenanthe leucura	No	1	No		30.37	2.32	10
Oenanthe lugens	No		No				
Oenanthe moesta	No		No				
Oenanthe monacha	No		No				
Oenanthe oenanthe	No	1	No		15.8	22.42	8800
Oenanthe pleschanka	No		No				
Oenanthe xanthoprymna	No		No				
Oriolus oriolus	No	5	No		39.67	15.49	5250
Otis tarda	No	2	No				

Otus scops	No		Yes				
Oxyura leucocephala	No		No				
Pandion haliaetus	No	19	No				
Panurus biarmicus	No		No				
Parus ater	Yes	3	No		5.55	17.46	20500
Parus caeruleus	Yes	11	Yes		5.54	18.84	32000
Parus cinctus	No		No				
Parus cristatus	No	1	No		6.32	16.27	9050
Parus cyanus	No		No				
Parus lugubris	No	1	No				
Parus major	Yes	14	Yes		5.22	18.71	68500
Parus montanus	Yes	1	No				
Parus palustris	Yes	3	No		5.59	15.96	4500
Passer domesticus	Yes	41	Yes	0.8	3.83	19.22	96500
Passer hispaniolensis	No		No		11.94	8.60	4500
Passer iagoensis	No		No				
Passer moabiticus	No		No				
Passer montanus	Yes	5	Yes	0.6	5.11	18.34	37000
Pelagodroma marina	No	1	No				
Pelecanus crispus	No		No				
Pelecanus onocrotalus	No	3	No				
Perdix perdix	No	1	No	0.48	24.78	15.35	2350
Perisoreus infaustus	Yes		No				
Pernis apivorus	No	2	No				

Petronia brachydactyla	No		No				
Petronia petronia	No		No				
Petronia xanthocollis	No	1	No				
Phalacrocorax aristotelis	No	2	No				
Phalacrocorax carbo	Yes	7	No		75.29	22.45	340
Phalacrocorax pygmeus	No		No				
Phalaropus fulicarius	No		No				
Phalaropus lobatus	No	1	No				
Philomachus pugnax	No	3	No				
Phoenicopterus ruber	No	2	No				
Phoenicurus erythrogastrus	No		No				
Phoenicurus erythronotus	No		No				
Phoenicurus moussieri	No		No				
Phoenicurus ochruros	No	1	Yes		6.95	14.51	6400
Phoenicurus phoenicurus	No		Yes		9.1	18.04	11400
Phylloscopus bonelli	No		No		4.95	8.60	2450
Phylloscopus borealis	No		No				
Phylloscopus	No		No				

canariensis							
Phylloscopus collybita	No	7	Yes		6.49	20.36	45445
Phylloscopus inornatus	No	1	No				
Phylloscopus nitidus	No		No				
Phylloscopus sibilatrix	No		No				
Phylloscopus sindianus	No		No				
Phylloscopus trochiloides	No		No				
Phylloscopus trochilus	No	1	No		6.28	13.13	78000
Pica pica	No	20	Yes		14.37	19.42	13250
Picoides tridactylus	No		No				
Picus canus	No	2	No				
Picus viridis	No	7	Yes		17	15.59	945
Pinicola enucleator	Yes	1	No				
Platalea leucorodia	No		No				
Plectrophenax nivalis	No	1	No				
Plegadis falcinellus	No		No				
Pluvialis apricaria	No	1	No				
Pluvialis squatarola	No	1	No		51	1.54	7
Podiceps auritus	No	2	No				

Podiceps cristatus	No	2	No				
Podiceps grisegena	No	1	No				
Podiceps nigricollis	No	1	No				
Polysticta stelleri	No		No				
Porphyrio porphyrio	No	5	No				
Porzana parva	No		No				
Porzana porzana	No	1	No				
Porzana pusilla	No	1	No				
Prinia gracilis	No		No				
Prunella atrogularis	No		No				
Prunella collaris	No		No				
Prunella modularis	No	5	Yes	1	5.08	17.19	19000
Prunella montanella	No		No				
Prunella ocularis	No		No				
Pterocles alchata	No		No				
Pterocles orientalis	No		No				
Pterodroma feae	No		No				
Pterodroma madeira	No		No				
Puffinus assimilis	No	1	No				
Puffinus mauretanicus	No		No				
Puffinus puffinus	No	3	No				
Puffinus yelkouan	No		No				
Pycnonotus leucogenys	No		No				

Pyrrhocorax graculus	No	1	No				
Pyrrhocorax pyrrhocorax	No	6	No				
Pyrrhula murina	No		No				
Pyrrhula pyrrhula	No	5	No	0	6.01	18.40	10650
Rallus aquaticus	No	8	No				
Recurvirostra avosetta	No	2	No				
Regulus ignicapilla	No		No		5.25	9.43	5000
Regulus madeirensis	No		No				
Regulus regulus	No	3	No		4.49	19.97	27000
Remiz pendulinus	No	1	No		6.17	13.99	315
Rhamphocoris clotbey	No		No				
Rhodopechys obsoletus	No		No				
Rhodopechys sanguineus	No		No				
Riparia riparia	No	7	No		23.1	18.44	7450
Rissa tridactyla	Yes	3	No				
Saxicola dacotiae	No		No				
Saxicola rubetra	No	2	No		16.65	16.61	7700
Saxicola torquatus	No		No		9.27	17.35	3300
Scolopax rusticola	No	2	No				
Scotocerca inquieta	No		No				

Serinus canaria	No		No				
Serinus citrinelloides	No		No				
Serinus pusillus	No		No				
Serinus serinus	No		Yes		5.84	12.82	14150
Serinus syriacus	No		No				
Sitta europaea	Yes		No		6.59	17.00	13250
Sitta krueperi	No		No				
Sitta ledanti	No		No				
Sitta neumayer	No		No				
Sitta tephronota	No		No				
Sitta whiteheadi	No		No				
Somateria mollissima	No	1	No				
Somateria spectabilis	No		No				
Stercorarius longicaudus	No	3	No				
Stercorarius parasiticus	No	1	No				
Stercorarius pomarinus	No	5	No				
Sterna albifrons	No	1	No				
Sterna caspia	No	2	No				
Sterna dougallii	No		No				
Sterna hirundo	No	15	No				
Sterna nilotica	No	4	No				
Sterna paradisaea	No	2	No				

Sterna sandvicensis	No	3	No				
Stigmatopelia senegalensis	No		No				
Streptopelia decaocto	Yes	3	Yes	1	5.38	18.10	7850
Streptopelia turtur	No	1	No	0			
Strix aluco	No		Yes				
Strix nebulosa	No		No				
Strix uralensis	No		No				
Sturnus roseus	No		No				
Sturnus unicolor	No		Yes		14.3	2.13	2600
Sturnus vulgaris	Yes	24	Yes	0.64	9.75	21.87	39500
Surnia ulula	No		No				
Sylvia atricapilla	No	9	Yes	0	5.84	22.05	37000
Sylvia borin	No		No		6.6	16.64	24000
Sylvia cantillans	No		No		5.69	4.15	2300
Sylvia communis	No	2	No	0	8.15	18.09	19500
Sylvia conspicillata	No		No		7.27	6.41	310
Sylvia curruca	No	1	Yes		5.28	16.74	6300
Sylvia deserticola	No		No				
Sylvia hortensis	No		No				
Sylvia leucomelaena	No		No				
Sylvia melanocephala	No		No		6.64	5.64	5600
Sylvia melanothorax	No		No				
Sylvia mystacea	No		No				
Sylvia nana	No		No				

Sylvia nisoria	No		No				
Sylvia rueppelli	No		No				
Sylvia sarda	No		No				
Sylvia undata	No		No		10.16	4.03	2800
Tachybaptus ruficollis	No	7	No				
Tachymarptis melba	No	1	Yes				
Tadorna ferruginea	No		No				
Tadorna tadorna	No		No		36.3	17.04	54
Tarsiger cyanurus	No		No				
Tetrao mlokosiewiczi	No		No				
Tetrao tetrix	No		No	0			
Tetrao urogallus	No		No				
Tetraogallus caspius	No		No				
Tetraogallus caucasicus	No		No				
Tetrax tetrax	No		No				
Tichodroma muraria	No		No				
Tringa erythropus	No	2	No				
Tringa glareola	No		No				
Tringa nebularia	No	6	No		30	8.96	118
Tringa ochropus	No		No				
Tringa stagnatilis	No	1	No				
Tringa totanus	No	2	No		29.71	22.23	445
Troglodytes	No	1	Yes		5.64	21.61	31500

troglodytes							
Turdoides altirostris	No		No				
Turdoides squamiceps	No		No				
Turdus iliacus	No	4	Yes		12.75	11.45	18500
Turdus merula	Yes	29	Yes	0.57	7.11	22.42	61000
Turdus philomelos	No	10	Yes	0.38	7.73	17.68	28000
Turdus pilaris	No	5	Yes		14.21	11.24	19000
Turdus ruficollis	No		No				
Turdus torquatus	No		No				
Turdus viscivorus	No	4	No		19.33	17.98	5200
Tyto alba	No		Yes				
Upupa epops	No		No		19.96	16.14	1295
Uria aalge	No	3	No				
Uria lomvia	No		No				
Vanellus vanellus	No	1	No	0	34.69	17.78	2250
Xenus cinereus	No		No				

R Script

```
install.packages("ape"); library(ape)

t<-read.tree("tree.txt")
d<-read.csv("Supplementary Table S1.csv",header=T,row.name=1)

d[,2]<-log10(d[,2])
d[,5]<-log10(d[,5])
d[,6]<-log10(d[,6])
d[,7]<-log10(d[,7]/1000)

###Logistic Regression
for (i in 2:dim(d)[2]){
d1<-d[complete.cases(d[,i]),c(1,i)]
d1<-as.data.frame(d1)
glm.fit <- glm(Feeding_from_hand~.,data=d1, family = binomial)
print(summary(glm.fit))
}

###Phylogenetic Regression
for (i in 2:dim(d)[2]){
d1<-d[complete.cases(d[,i]),c(1,i)]
drop<-setdiff(t$tip.label,rownames(d1))
t1<-drop.tip(t,drop)
d1<-as.data.frame(d1)
model<-binaryPGLMM(Feeding_from_hand~.,data=d1,t1)
print(model)
}
```

Tree-txt

((((Francolinus_francolinus:20.425762,((((Lagopus_muta:3.043972,Lagopus_lagopus:3.043972):4.198749,((Tetrao_tetrix:1.651343,Tetrao_mlokosiewiczi:1.651343):2.358775,Tetrao_urogallus:4.010118):3.232603):3.916615,Bonasa_bonasia:11.159336):5.213256,Perdix_perdix:16.372592):4.05317):0.66371,((((Alectoris_graeca:2.722472,(Alectoris_rufa:1.661545,Alectoris_chukar:1.661545):1.060927):3.34036,Alectoris_barbara:6.062832):10.178932,(Tetraogallus_caspius:3.380261,Tetraogallus_caucasicus:3.380261):12.861503):1.455244,Coturnix_coturnix:17.697008):3.392465):57.162131,((((((Anas_penelope:2.600628,Anas_strepera:2.600628):2.440602,((Anas_crecca:3.48534,Anas_acuta:3.48534):0.273408,Anas_platyrhynchos:3.758748):1.282482):1.491106,(Anas_querquedula:3.325863,Anas_clypeata:3.325863):3.206473):4.228121,((((((Aythya_fuligula:1.212669,Aythya_marila:1.212669):0.61903,Aythya_ferina:1.831699):0.447535,Aythya_nyroca:2.279234):0.540906,Netta_rufina:2.82014):4.254679,Marmaronetta_angustirostris:7.074819):2.784749,(((((Mergellus_albellus:5.514054,(Mergus_merganser:1.128163,Mergus_serrator:1.128163):4.385891):1.351756,(Bucephala_islandica:1.076035,Bucephala_clangula:1.076035):5.789776):0.695946,(Melanitta_nigra:5.972869,Melanitta_fusca:5.972869):1.588887):1.363095,(Clangula_hyemalis:7.999732,(Histrionicus_histrionicus:7.24625,(Polysticta_stelleri:4.804469,(Somateria_spectabilis:0.286362,Somateria_mollissima:0.286362):4.518107):2.441781):0.753483):0.925119):0.590028,(Tadorna_tadorna:2.456289,Tadorna_ferruginea:2.456289):7.05859):0.344689):0.900888):9.307655,Oxyura_leucocephala:20.068112):1.25581,(((Branta_bernicla:5.325741,Branta_leucopsis:5.325741):2.505921,((Anser_albifrons:0.827047,Anser_fabalis:0.827047):0.773596,((Anser_brachyrhynchus:0.743974,Anser_anser:0.743974):0.113796,Anser_erythropus:0.85777):0.742873):6.231019):3.867036,((Cygnus_cygnus:1.685174,Cygnus_columbianus:1.685174):5.919692,Cygnus_olor:7.604866):4.093832):9.625224):56.927681):21.667294,((((((Tachybaptus_ruficollis:37.670479,((Podiceps_auritus:17.621947,(Podiceps_grisegena:15.630383,Podiceps_cristatus:15.630383):1.991563):4.809264,Podiceps_nigricollis:22.431211):15.239268):16.439348,Phoenicopterus_ruber:54.109827):19.491679,((Pterocles_alchata:30.955105,Pterocles_orientalis:30.955105):42.925451,(((Columba_junoniae:14.836706,((Columba_bollii:3.473196,Columba_palumbus:3.473196):7.474518,(Columba_livia:7.394334,Columba_oenas:7.394334):3.55338):3.888991):0.785143,Columba_trocaz:15.621849):2.125433,((Streptopelia_decaocto:9.599213,Streptopelia_turtur:9.599213):7.370719,Stigmatopelia_senegalensis:16.969933):0.777349):56.133275):0.01):3.088706,((((((((Porzana_pusilla:15.99915,Fulica_cristata:15.99915):13.26392,((Porzana_porzana:14.09836,Porzana_parva:14.09836):8.65347,(Gallinula_chloropus:13.829245,Fulica_atra:13.829245):8.922584):6.51124):2.092669,(Crex_crex:18.637887,Rallus_aquaticus:18.637887):12.717852):2.635899,Porphyrio_porphyrio:33.991638):17.74803,(Grus_grus:6.6886,Grus_virgo:6.6886):45.051067):15.943675,((Chlamydotis_undulata:14.614693,Otis_tarda:14.614693):16.11324,Tetrax_tetrax:30.727933):36.95541):1.828278,((Cuculus_saturatus:1.827105,Cuculus_canorus:1.827105):45.39594,Clamator_glandarius:47.223046):22.288575):3.281018,((Gavia_stellata:47.460275,(Gavia_arctica:19.482201,Gavia_immer:19.482201):27.978074):19.871742,(((((((Platalea_leucorodia:46.22209,Plegadis_falcinellus:46.22209):7.163715,Geronticus_eremita:53.385804):6.683385,(((Egretta_garzetta:30.69889,(Ardeola_ralloides:26.928908,Nycticorax_nycticorax:26.928908):3.769982):2.03559,((Bubulcus_ibis:16.291986,(Ardea_purpurea:12.33813,Ardea_cinerea:12.33813):3.953855):2.017912,Casmerodius_albus:18.309898):14.424581):6.821721,(Ixobrychus_minutus:25.04248,Botaurus_stellaris:25.04248):14.513721):20.512989):2.674036,(Pelecanus_onocrotalus:15.883984,Pelecanus_crispus:15.883984):46.859242):1.69332,(Morus_bassanus:42.507883,(Phalacrocorax_

aristotelis:13.363262,(Phalacrocorax_pygmeus:10.005806,Phalacrocorax_carbo:10.005806):
3.357456):29.144621):21.928662):1.248237,(Ciconia_nigra:22.303689,Ciconia_ciconia:22.30
3689):43.381093):0.924747,((((Pterodroma_madeira:2.871983,Pterodroma_feae:2.871983):
36.747195,((Bulweria_bulwerii:30.868132,(Calonectris_diomedea:20.36169,((Puffinus_maur
etanicus:3.564119,Puffinus_yelkouan:3.564119):3.967932,(Puffinus_puffinus:5.351302,Puffi
nus_assimilis:5.351302):2.180749):12.829639):10.506442):5.279583,Fulmarus_glacialis:36.1
47715):3.471464):15.132597,Pelagodroma_marina:54.751776):5.729056,(Oceanodroma_ca
stro:35.52731,(Oceanodroma_leucorhoa:30.948573,Hydrobates_pelagicus:30.948573):4.57
8737):24.953522):6.128699):0.722486):5.460623):3.897573):5.706258,((((((((Larus_minutus:
5.954967,((Larus_genei:3.138361,Larus_ridibundus:3.138361):1.347749,((Larus_canus:1.418
361,(((Larus_glaucoides:0.284844,Larus_cachinnans:0.284844):0.248379,Larus_fuscus:0.533
223):0.243972,((Larus_hyperboreus:0.203642,Larus_argentatus:0.203642):0.420295,(Larus_
marinus:0.318438,Larus_armenicus:0.318438):0.305499)):0.153258):0.641166):1.405759,((L
arus_melanocephalus:1.131636,Larus_ichthyaetus:1.131636):0.316394,Larus_audouinii:1.4
4803):1.37609):1.66199):1.468857):0.301612,Rissa_tridactyla:6.256578):11.815189,(Sterna_
albifrons:11.465241,((Sterna_nilotica:6.39123,Sterna_caspia:6.39123):2.279793,((Sterna_sa
ndvicensis:5.621836,((Sterna_dougallii:3.163387,Sterna_hirundo:3.163387):0.364098,Sterna
_paradisaea:3.527485):2.094351):1.807516,((Chlidonias_leucopterus:1.439026,Chlidonias_n
iger:1.439026):1.803736,Chlidonias_hybrida:3.242762):4.186591):1.24167):2.794218):6.606
526):2.467012,(((Stercorarius_longicaudus:4.123447,Stercorarius_parasiticus:4.123447):3.0
66162,(Stercorarius_pomarinus:0.415428,Catharacta_skua:0.415428):6.774181):11.552606,
((((Uria_lomvia:4.059568,Uria_aalge:4.059568):5.85629,(Alle_alle:9.051604,Alca_torda:9.05
1604):0.864254):3.396331,Cepphus_grylle:13.312189):2.208277,Fratercula_arctica:15.5204
65):3.22175):1.796564):4.022179,(Cursorius_cursor:19.10866,(Glareola_pratincola:0.928107
,Glareola_nordmanni:0.928107):18.180552):5.452299):32.607761,((((Arenaria_interpres:27.
201123,(((Limicola_falcinellus:15.237513,Philomachus_pugnax:15.237513):2.647379,(((Calid
ris_maritima:9.123065,Calidris_alpina:9.123065):4.243166,(Calidris_alba:7.401921,Calidris_
minuta:7.401921):5.96431):2.127873,Calidris_temminckii:15.494104):2.390789):0.299956,C
alidris_canutus:18.184849):9.016274):6.301703,(((Xenus_cinereus:25.649329,(Phalaropus_l
obatus:6.220801,Phalaropus_fulicarius:6.220801):19.428528):3.005189,((((Tringa_erythropu
s:11.209446,Tringa_nebularia:11.209446):3.275792,(Tringa_glareola:8.272426,(Tringa_totan
us:7.358764,Tringa_stagnatilis:7.358764):0.913663):6.212812):5.277236,Tringa_ochropus:1
9.762474):7.33694,Actitis_hypoleucos:27.099414):1.555105):3.615602,(Scolopax_rusticola:
26.547587,((Gallinago_stenura:7.339981,Gallinago_media:7.339981):5.448206,Gallinago_ga
llinago:12.788186):13.759401):5.722533):1.232705):2.577406,(Lymnocryptes_minimus:29.4
39759,(Limosa_lapponica:13.786874,Limosa_limosa:13.786874):15.652885):6.640472):3.92
4913,(Numenius_phaeopus:14.589921,Numenius_arquata:14.589921):25.415224):17.16357
5):7.17373,(((((Charadrius_alexandrinus:34.071875,Vanellus_vanellus:34.071875):2.695445,
((Charadrius_dubius:24.355389,Charadrius_hiaticula:24.355389):5.971947,Eudromias_mori
nellus:30.327336):6.439985):7.259896,((Recurvirostra_avosetta:18.914223,Himantopus_hi
mantopus:18.914223):9.575735,Haematopus_ostralegus:28.489958):15.537259):3.231568,(
Pluvialis_squatarola:9.233559,Pluvialis_apricaria:9.233559):38.025225):11.916665,Burhinus
_oedicnemus:59.175449):5.167001):17.716572,((((Tyto_alba:64.81986,(((((Bubo_scandiaca:
12.720444,Bubo_bubo:12.720444):11.510402,((Strix_aluco:8.517814,Strix_uralensis:8.5178
14):3.049347,Strix_nebulosa:11.567161):12.663686):6.000133,(Asio_flammeus:13.416906,A
sio_otus:13.416906):16.814074):3.92507,Otus_scops:34.15605):7.15257,(((Surnia_ulula:25.
824454,Glaucidium_passerinum:25.824454):3.835558,Athene_noctua:29.660012):2.587372,

Aegolius_funereus:32.247384):9.061236):23.511239):12.984195,((((Coracias_garrulus:60.54
8364,Alcedo_atthis:60.548364):0.954503,Merops_apiaster:61.502867):7.671627,(((Dryocop
us_martius:11.315817,(Picus_viridis:3.503113,Picus_canus:3.503113):7.812704):4.918564,((
(((Dendrocopos_major:1.545351,Dendrocopos_syriacus:1.545351):0.842505,Dendrocopos_l
eucotos:2.387856):7.902721,Dendrocopos_minor:10.290577):0.619756,Dendrocopos_medi
us:10.910333):2.258623,Picoides_tridactylus:13.168956):3.065426):11.186301,Jynx_torquill
a:27.420682):41.753811):1.853847,Upupa_epops:71.028341):6.775714):1.370163,((((Pernis
_apivorus:35.847221,(Gypaetus_barbatus:24.88937,Neophron_percnopterus:24.88937):10.
957851):5.717593,(((Aegypius_monachus:15.179856,Gyps_fulvus:15.179856):14.82074,Circ
aetus_gallicus:30.000596):4.162482,((((((((Circus_macrourus:6.877525,Circus_aeruginosus:6
.877525):4.05253,Circus_cyaneus:10.930055):1.18198,Circus_pygargus:12.112035):8.97957
7,Accipiter_gentilis:21.091612):1.855275,Accipiter_nisus:22.946887):1.931331,Accipiter_bre
vipes:24.878217):4.132279,(((Milvus_milvus:1.57985,Milvus_migrans:1.57985):18.064868,H
aliaeetus_albicilla:19.644718):3.561124,((Buteo_rufinus:0.758518,Buteo_buteo:0.758518):1
.711123,Buteo_lagopus:2.469641):20.736201):5.804654):3.432838,((((Aquila_chrysaetos:7.4
52159,Aquila_fasciatus:7.452159):1.572117,Hieraaetus_pennatus:9.024276):0.525365,((Aqu
ila_adalberti:1.68039,Aquila_heliaca:1.68039):2.196337,Aquila_nipalensis:3.876728):5.6729
13):1.249923,(Aquila_clanga:1.984334,Aquila_pomarina:1.984334):8.81523):21.643771):1.7
19744):7.401736):13.431143,Elanus_caeruleus:54.995957):4.87923,Pandion_haliaetus:59.8
75186):19.299031):1.181835,((((((Falco_eleonorae:2.620517,Falco_subbuteo:2.620517):7.3
47151,Falco_vespertinus:9.967668):0.980726,((Falco_biarmicus:1.868943,(Falco_cherrug:1.
321027,Falco_rusticolus:1.321027):0.547916):2.154763,Falco_peregrinus:4.023706):6.9246
88):1.251167,(Falco_naumanni:7.769054,Falco_tinnunculus:7.769054):4.430507):1.038899,
Falco_columbarius:13.23846):64.962833,((Oriolus_oriolus:30.736198,(((Pyrrhocorax_pyrrho
corax:9.801876,Pyrrhocorax_graculus:9.801876):11.629082,((Cyanopica_cyanus:17.656122,
Perisoreus_infaustus:17.656122):1.469149,(((Nucifraga_caryocatactes:12.942181,(((Corvus_
corax:5.751714,Corvus_frugilegus:5.751714):1.313866,(Corvus_ruficollis:4.064819,(Corvus_
cornix:0.01,Corvus_corone:0.01):4.054819):3.000761):3.120396,Corvus_monedula:10.18597
7):2.756204):2.171576,Pica_pica:15.113757):1.036421,Garrulus_glandarius:16.150178):2.97
5093):2.305687):4.829896,((Lanius_nubicus:9.206134,((Lanius_isabellinus:2.221554,Lanius_
collurio:2.221554):6.211774,(Lanius_minor:4.899016,Lanius_senator:4.899016):3.534312):0
.772806):0.774923,Lanius_excubitor:9.981056):16.279797):4.475345):19.630751,(((((((((Tur
doides_altirostris:6.413646,Turdoides_squamiceps:6.413646):14.719253,((Sylvia_atricapilla:
10.828452,Sylvia_borin:10.828452):3.663539,(Sylvia_nana:11.191765,((Sylvia_nisoria:9.673
281,(Sylvia_curruca:5.970832,(Sylvia_hortensis:3.649613,Sylvia_leucomelaena:3.649613):2.
321219):3.702449):0.691396,(((Sylvia_melanothorax:4.494531,Sylvia_rueppelli:4.494531):1.
625622,(Sylvia_cantillans:4.045819,(Sylvia_mystacea:2.766018,Sylvia_melanocephala:2.766
018):1.279801):2.074334):0.929233,((Sylvia_conspicillata:5.316128,((Sylvia_deserticola:1.75
5706,Sylvia_undata:1.755706):1.6366,Sylvia_sarda:3.392306):1.923823):0.39373,Sylvia_com
munis:5.709859):1.339527):3.315291):0.827088):3.300227):6.640908):3.626072,(((Acroceph
alus_palustris:17.564012,(((Locustella_luscinioides:5.634382,Locustella_fluviatilis:5.634382):
3.199837,Locustella_lanceolata:8.834219):0.601195,Locustella_naevia:9.435413):8.128598):
8.922087,((((((Acrocephalus_dumetorum:6.09468,Acrocephalus_scirpaceus:6.09468):0.8721
63,Acrocephalus_agricola:6.966844):2.701202,((Acrocephalus_schoenobaenus:4.926463,Acr
ocephalus_melanopogon:4.926463):2.061257,Acrocephalus_paludicola:6.987719):2.680326
):0.753916,(Acrocephalus_brevipennis:5.914006,(Acrocephalus_arundinaceus:2.816193,Acr
ocephalus_stentoreus:2.816193):3.097814):4.507956):1.540685,(Hippolais_caligata:8.10454

9,Hippolais_pallida:8.104549):3.858098):1.110203,((Hippolais_icterina:4.467945,Hippolais_
polyglotta:4.467945):3.152973,(Hippolais_languida:5.972382,Hippolais_olivetorum:5.97238
2):1.648535):5.451932):13.413249):1.068904,Scotocerca_inquieta:27.555003):0.01):3.3178
77,(Prinia_gracilis:20.067392,Cisticola_juncidis:20.067392):8.009456):3.462157,Pycnonotus
_leucogenys:31.539005):1.925023,((((((Phylloscopus_sibilatrix:4.940257,Phylloscopus_bonel
li:4.940257):9.984958,((((Phylloscopus_canariensis:1.232775,Phylloscopus_sindianus:1.2327
75):1.12295,Phylloscopus_collybita:2.355725):3.175572,Phylloscopus_trochilus:5.531298):8.
632859,Phylloscopus_inornatus:14.164157):0.761058):2.101295,(Phylloscopus_borealis:10.
50534,(Phylloscopus_trochiloides:2.23595,Phylloscopus_nitidus:2.23595):8.269389):6.52117
):5.023743,Cettia_cetti:22.050253):2.06309,Aegithalos_caudatus:24.113342):5.563655,(Ripa
ria_riparia:18.819544,(((Hirundo_rupestris:13.848107,Hirundo_rustica:13.848107):1.83374,
Hirundo_daurica:15.681847):0.411405,Delichon_urbicum:16.093252):2.726292):10.857453)
:3.787031):3.747335,(Panurus_biarmicus:28.188635,(((Alaemon_alaudipes:20.31451,(Amm
omanes_deserti:15.123828,Rhamphocoris_clotbey:15.123828):5.190682):1.874522,((((((Ala
uda_arvensis:9.148409,(Galerida_cristata:5.030157,Galerida_theklae:5.030157):4.118252):1
.611011,Lullula_arborea:10.759419):1.829272,(((Melanocorypha_yeltoniensis:3.837247,Mel
anocorypha_calandra:3.837247):5.441782,(Melanocorypha_bimaculata:3.596099,Melanoco
rypha_leucoptera:3.596099):5.68293):2.232669,Eremalauda_dunni:11.511697):1.076995):0.
61009,(Calandrella_brachydactyla:11.686337,Calandrella_rufescens:11.686337):1.512445):1
.049836,(Eremophila_alpestris:2.601596,Eremophila_bilopha:2.601596):11.647022):2.50475
1,Chersophilus_duponti:16.753368):5.435664):1.474034,(Ammomanes_cinctura:15.785344,
Eremopterix_nigriceps:15.785344):7.877722):4.525569):9.022728):4.914422,((((((Parus_lug
ubris:12.204283,(Parus_cinctus:10.602368,Parus_montanus:10.602368):1.601916):2.502254
,Parus_ater:14.706537):0.541156,Parus_cristatus:15.247692):1.022054,(Parus_major:8.129
166,Parus_palustris:8.129166):8.14058):2.654536,(Parus_caeruleus:2.65228,Parus_cyanus:2
.65228):16.272002):17.491837,Remiz_pendulinus:36.416119):5.709666):2.381334,((((Regul
us_madeirensis:14.77651,Regulus_ignicapilla:14.77651):14.660426,Regulus_regulus:29.436
936):13.589124,(((((Tichodroma_muraria:25.487527,(Certhia_brachydactyla:7.094626,Certh
ia_familiaris:7.094626):18.392902):3.49692,Troglodytes_troglodytes:28.984447):3.596012,((
((Sitta_krueperi:1.605513,Sitta_ledanti:1.605513):5.405643,Sitta_whiteheadi:7.011156):7.1
44668,Sitta_europaea:14.155824):5.773814,(Sitta_tephronota:0.590783,Sitta_neumayer:0.5
90783):19.338855):12.65082):6.308819,((((((((Turdus_torquatus:3.475034,Turdus_ruficollis:
3.475034):2.195021,Turdus_pilaris:5.670056):3.840213,Turdus_merula:9.510269):0.490479,
Turdus_iliacus:10.000748):3.781363,(Turdus_philomelos:12.052456,Turdus_viscivorus:12.05
2456):1.729655):12.281428,(Tarsiger_cyanurus:18.729348,((((Irania_gutturalis:14.293259,((
Luscinia_megarhynchos:2.938028,Luscinia_luscinia:2.938028):10.065607,Luscinia_svecica:1
3.003635):1.289624):2.180212,((((((Saxicola_rubetra:8.996708,(Saxicola_torquatus:3.73109
2,Saxicola_dacotiae:3.731092):5.265616):3.947746,((((Oenanthe_deserti:5.613431,Oenanth
e_monacha:5.613431):0.939295,(Oenanthe_pleschanka:0.214231,Oenanthe_hispanica:0.21
4231):6.338495):1.333917,(Oenanthe_oenanthe:4.434654,Oenanthe_isabellina:4.434654):3
.451989):1.805047,((Cercomela_melanura:6.749792,(((Oenanthe_leucopyga:3.426244,Oena
nthe_albonigra:3.426244):0.459064,Oenanthe_leucura:3.885308):0.329134,((Oenanthe_lug
ens:0.862967,Oenanthe_finschii:0.862967):0.766343,Oenanthe_xanthoprymna:1.62931):2.5
85132):2.53535):0.570546,Oenanthe_moesta:7.320339):2.371351):3.252765):0.856942,(Mo
nticola_solitarius:2.618556,Monticola_saxatilis:2.618556):11.18284):0.786193,((((Phoenicur
us_moussieri:5.560268,Phoenicurus_phoenicurus:5.560268):0.061304,Phoenicurus_ochruro
s:5.621572):0.825604,Phoenicurus_erythrogastrus:6.447176):4.840067,Phoenicurus_erythr

onotus:11.287243):3.300346):0.907695,Luscinia_calliope:15.495284):0.373051,(((Ficedula_h
ypoleuca:1.58359,Ficedula_albicollis:1.58359):0.56565,Ficedula_semitorquata:2.14924):6.23
3348,Ficedula_parva:8.382588):7.485747):0.605136):0.383424,Erithacus_rubecula:16.85689
5):1.281615,(Muscicapa_striata:14.412087,Erythropygia_galactotes:14.412087):3.726423):0
.590838):7.33419):2.649345,Cinclus_cinclus:28.712884):0.852802,((Sturnus_unicolor:0.2678
27,Sturnus_vulgaris:0.267827):8.144669,Sturnus_roseus:8.412495):21.15319):9.323592):2.3
84358,(Bombycilla_garrulus:31.286952,Hypocolius_ampelinus:31.286952):9.986684):1.7524
24):0.567387,((((((((Fringilla_teydea:5.046798,Fringilla_coelebs:5.046798):2.943009,Fringilla
_montifringilla:7.989807):17.951051,(((((Pyrrhula_pyrrhula:4.622897,Pyrrhula_murina:4.622
897):7.677407,Pinicola_enucleator:12.300304):4.728991,((Eremopsaltria_mongolicus:6.500
312,Bucanetes_githagineus:6.500312):9.761214,((Carpodacus_erythrinus:11.665256,Carpod
acus_synoicus:11.665256):2.804425,Carpodacus_rubicilla:14.46968):1.791845):0.76777):0.8
63813,((((Carduelis_hornemanni:0.618521,Carduelis_flammea:0.618521):4.966293,(((Loxia_
curvirostra:0.417879,Loxia_pytyopsittacus:0.417879):0.226285,Loxia_scotica:0.644164):1.13
5503,Loxia_leucoptera:1.779667):3.805147):3.56427,((((Carduelis_flavirostris:4.166051,Card
uelis_cannabina:4.166051):2.925176,Carduelis_spinus:7.091226):1.466221,((Serinus_syriacu
s:7.184646,Serinus_citrinelloides:7.184646):1.283144,((Serinus_canaria:5.537399,Serinus_p
usillus:5.537399):0.927018,Serinus_serinus:6.464417):2.003373):0.089658):0.383561,Cardu
elis_carduelis:8.941009):0.208076):2.173413,((Rhodopechys_obsoletus:2.079296,Rhodopec
hys_sanguineus:2.079296):6.045786,Carduelis_chloris:8.125082):3.197415):6.570611):2.596
263,Coccothraustes_coccothraustes:20.489372):5.451486):1.475073,((Calcarius_lapponicus:
18.391367,Plectrophenax_nivalis:18.391367):5.388077,(((((((Emberiza_cirlus:6.003566,(Emb
eriza_leucocephalos:0.405868,Emberiza_citrinella:0.405868):5.597697):1.841608,((Emberiz
a_caesia:2.389311,Emberiza_hortulana:2.389311):3.501195,Emberiza_buchanani:5.890506)
:1.954668):1.080812,Emberiza_cia:8.925985):1.023849,Miliaria_calandra:9.949835):2.22787
3,(Emberiza_melanocephala:4.389055,Emberiza_bruniceps:4.389055):7.788652):0.686506,((
Emberiza_pallasi:4.663397,Emberiza_schoeniclus:4.663397):3.870967,(Emberiza_rustica:7.0
96509,(Emberiza_aureola:5.328077,Emberiza_pusilla:5.328077):1.768432):1.437855):4.329
85):1.006942,(Emberiza_striolata:6.912303,Emberiza_cineracea:6.912303):6.958853):9.908
288):3.636487):3.026382,((((Anthus_berthelotii:2.31571,Anthus_campestris:2.31571):3.522
35,Anthus_novaeseelandiae:5.83806):12.814567,((((Anthus_pratensis:3.85233,Anthus_spin
oletta:3.85233):2.624211,Anthus_cervinus:6.476541):4.927768,(Anthus_hodgsoni:3.856873,
Anthus_trivialis:3.856873):7.547436):2.776295,Anthus_gustavi:14.180604):4.472022):3.687
888,(((Motacilla_flava:2.07363,Motacilla_citreola:2.07363):1.417577,Motacilla_alba:3.4912
07):0.926415,Motacilla_cinerea:4.417622):17.922893):8.101798):1.5568,(((Petronia_petroni
a:16.239569,Montifringilla_nivalis:16.239569):5.164054,((Passer_moabiticus:7.70278,(Passe
r_montanus:6.728714,(Passer_domesticus:2.614865,Passer_hispaniolensis:2.614865):4.113
849):0.974066):2.771404,Passer_iagoensis:10.474184):10.929439):6.234925,((Carpospiza_b
rachydactyla:0.01,Petronia_brachydactyla:0.01):7.492426,Petronia_xanthocollis:7.502426):2
0.136121):4.360565):3.523936,((((Prunella_collaris:0.266874,Prunella_modularis:0.266874):
4.859402,Prunella_montanella:5.126276):1.857184,Prunella_atrogularis:6.98346):4.74139,P
runella_ocularis:11.72485):23.798198):3.891217,((Anthreptes_metallicus:16.311226,Anthre
ptes_platurus:16.311226):3.082135,Nectarinia_osea:19.393361):20.020903):4.179183):0.91
3672):5.85983):27.834344):2.15476):1.702969):0.337448):0.595099,((Tachymarptis_melba:
16.142813,(((Apus_apus:4.229162,Apus_unicolor:4.229162):3.596965,Apus_affinis:7.82612
6):4.789249,(Apus_pallidus:5.827547,Apus_caffer:5.827547):6.787829):3.527438):59.90909

5,(Caprimulgus_europaeus:30.892073,Caprimulgus_ruficollis:30.892073):45.159835):6.9396
61):16.927327);

Supplementary Table S1.txt

Species,Feeding_from_hand,Number_of_innovation,Urban_tolerance,Introduction_success (%),Flight initiation distance (m),Breeding_range (million km2),Population_size (thousand)
Accipiter_brevipes,0,,0,,,,
Accipiter_gentilis,0,13,0,,,,
Accipiter_nisus,0,8,0,,10,20.20662,395
Acrocephalus_agricola,0,,0,,,,
Acrocephalus_arundinaceus,0,2,0,,,,
Acrocephalus_brevipennis,0,,0,,,,
Acrocephalus_dumetorum,0,,0,,,,
Acrocephalus_melanopogon,0,,0,,,,
Acrocephalus_paludicola,0,,0,,,,
Acrocephalus_palustris,0,,0,,8.84,12.98658,5000
Acrocephalus_schoenobaenus,0,2,0,,7.56,16.946488,5900
Acrocephalus_scirpaceus,0,,0,,6.73,16.341654,3850
Acrocephalus_stentoreus,0,,0,,,,
Actitis_hypoleucos,0,6,0,,17,18.567443,1160
Aegithalos_caudatus,0,7,1,,5.02,18.276019,8500
Aegolius_funereus,0,,0,,,,
Aegypius_monachus,0,,0,,,,
Alaemon_alaudipes,0,,0,,,,
Alauda_arvensis,0,5,0,0.64,31.37,18.626661,60000
Alca_torda,0,,0,,,,
Alcedo_atthis,0,4,0,,16.27,14.915965,120
Alectoris_barbara,0,,0,,,,
Alectoris_chukar,0,,0,,,,
Alectoris_graeca,0,2,0,,,,
Alectoris_rufa,0,3,0,0.09,35.54,2.543626,3250
Alle_alle,0,,0,,,,
Ammomanes_cinctura,0,,0,,,,
Ammomanes_deserti,0,,0,,,,
Anas_acuta,0,2,0,,,,
Anas_clypeata,0,,0,,,,
Anas_crecca,0,,0,,39.23,21.256084,1060
Anas_penelope,0,4,0,,,,
Anas_platyrhynchos,0,9,1,0.33,13.42,22.392575,4200
Anas_querquedula,0,3,0,,,,
Anas_strepera,0,,0,,,,
Anser_albifrons,0,3,0,,,,
Anser_anser,1,4,1,,,,
Anser_brachyrhynchus,0,,0,,,,
Anser_erythropus,0,2,0,,,,
Anser_fabalis,0,2,0,,,,
Anthreptes_metallicus,0,,0,,,,
Anthreptes_platurus,0,,0,,,,
Anthus_berthelotii,0,,0,,,,
Anthus_campestris,0,,0,,,,
Anthus_cervinus,0,,0,,,,
Anthus_gustavi,0,,0,,,,
Anthus_hodgsoni,0,,0,,,,
Anthus_novaeseelandiae,0,,0,,,,
Anthus_pratensis,0,2,0,,13.97,17.595781,11500
Anthus_spinoletta,0,3,0,,4.16,19.601138,1520
Anthus_trivialis,0,,0,,10.19,18.113965,34500
Apus_affinis,0,,1,,,,

Apus_apus,0,2,1,,38.1,18.290319,11950
Apus_caffer,0,,1,,,,
Apus_pallidus,0,,1,,,,
Apus_unicolor,0,2,1,,,,
Aquila_adalberti,0,,0,,,,
Aquila_chrysaetos,0,7,0,,,,
Aquila_clanga,0,,0,,,,
Aquila_fasciatus,0,,0,,,,
Aquila_heliaca,0,,0,,,,
Aquila_nipalensis,0,2,0,,,,
Aquila_pomarina,0,,0,,,,
Ardea_cinerea,1,26,1,,47.36,19.718995,250
Ardea_purpurea,0,3,0,,,,
Ardeola_ralloides,0,3,0,,,,
Arenaria_interpres,0,9,0,,20.5,3.182088,58
Asio_flammeus,0,,0,,,,
Asio_otus,0,,0,,,,
Athene_noctua,0,,0,1,36.43,14.334508,930
Aythya_ferina,0,,0,,,,
Aythya_fuligula,0,2,0,,10.68,18.581007,805
Aythya_marila,0,,0,,,,
Aythya_nyroca,0,,0,,,,
Bombycilla_garrulus,1,6,0,,,,
Bonasa_bonasia,0,,0,,,,
Botaurus_stellaris,0,3,0,,,,
Branta_bernicla,0,4,0,,23.5,4.409603,2
Branta_leucopsis,1,,0,,,,
Bubo_bubo,0,,0,,,,
Bubo_scandiaca,0,,0,,,,
Bubulcus_ibis,0,2,0,,24.38,8.949377,102
Bucanetes_githagineus,0,,0,,5.01,5.15657,16
Bucephala_clangula,0,,0,,,,
Bucephala_islandica,0,,0,,,,
Bulweria_bulwerii,0,,0,,,,
Burhinus_oedicnemus,0,2,0,,,,
Buteo_buteo,0,21,0,,54.06,20.992209,955
Buteo_lagopus,0,,0,,,,
Buteo_rufinus,0,,0,,,,
Calandrella_brachydactyla,0,,0,,,,
Calandrella_rufescens,0,,0,,,,
Calcarius_lapponicus,0,,0,,,,
Calidris_alba,0,6,0,,18,6.172932,38
Calidris_alpina,0,2,0,,,,
Calidris_canutus,0,3,0,,,,
Calidris_maritima,0,2,0,,,,
Calidris_minuta,0,2,0,,,,
Calidris_temminckii,0,,0,,,,
Calonectris_diomedea,0,2,0,,,,
Caprimulgus_europaeus,0,,0,,,,
Caprimulgus_ruficollis,0,,0,,,,
Carduelis_cannabina,0,3,1,0,9.25,18.741687,19000
Carduelis_carduelis,0,4,1,0.25,7.63,19.353063,20500
Carduelis_chloris,1,8,1,0.5,6.59,22.122015,23000
Carduelis_flammea,1,4,1,1,4.5,13.525065,13900
Carduelis_flavirostris,0,2,0,,,,
Carduelis_hornemanni,1,,0,,,,
Carduelis_spinus,0,7,0,0,4.83,18.127174,14000
Carpodacus_erythrinus,0,,0,,,,

Carpodacus_rubicilla,0,,0,,,,
Carpodacus_synoicus,0,,0,,,,
Carpospiza_brachydactyla,0,,0,,,,
Casmerodius_albus,0,2,0,,,,
Catharacta_skua,0,7,0,,,,
Cepphus_grylle,0,,0,,,,
Cercomela_melanura,0,,0,,,,
Certhia_brachydactyla,0,,0,,7.08,9.568575,6200
Certhia_familiaris,0,3,0,,,,
Cettia_cetti,0,2,0,,5.55,10.980957,1100
Charadrius_alexandrinus,0,,0,,,,
Charadrius_dubius,0,3,0,,,,
Charadrius_hiaticula,0,2,0,,19.65,15.177851,170
Chersophilus_duponti,0,,0,,,,
Chlamydotis_undulata,0,,0,,,,
Chlidonias_hybrida,0,,0,,,,
Chlidonias_leucopterus,0,2,0,,,,
Chlidonias_niger,0,7,0,,,,
Ciconia_ciconia,1,4,1,,,,
Ciconia_nigra,0,2,0,,,,
Cinclus_cinclus,0,5,0,,,,
Circaetus_gallicus,0,,0,,,,
Circus_aeruginosus,0,2,0,,,,
Circus_cyaneus,0,7,0,,,,
Circus_macrourus,0,,0,,,,
Circus_pygargus,0,6,0,,,,
Cisticola_juncidis,0,,0,,5.32,11.176228,665
Clamator_glandarius,0,,0,,,,
Clangula_hyemalis,0,2,0,,,,
Coccothraustes_coccothraustes,0,4,1,,,,
Columba_bollii,0,,0,,,,
Columba_junoniae,0,,0,,,,
Columba_livia,1,,1,,6.46,22.011569,12150
Columba_oenas,0,,0,,,,
Columba_palumbus,0,3,1,0,19.69,20.992209,13000
Columba_trocaz,0,,0,,,,
Coracias_garrulus,0,3,0,,,,
Corvus_corax,1,9,0,,79.63,24.716373,710
Corvus_cornix,0,33,1,,,,
Corvus_corone,0,33,1,,27.48,18.650578,12000
Corvus_frugilegus,0,12,1,0.75,44.39,16.762117,14000
Corvus_monedula,0,8,1,0,26.67,17.130066,10100
Corvus_ruficollis,0,,0,,,,
Coturnix_coturnix,0,,0,0,,,
Crex_crex,0,,0,,,,
Cuculus_canorus,0,3,0,,25.89,18.418485,6400
Cuculus_saturatus,0,,0,,,,
Cursorius_cursor,0,,0,,,,
Cyanopica_cyanus,0,,0,0,,,
Cygnus_columbianus,0,3,0,,,,
Cygnus_cygnus,0,2,0,,,,
Cygnus_olor,1,2,0,0.4,,,
Delichon_urbicum,0,8,1,,6.34,18.408794,16950
Dendrocopos_leucotos,0,2,0,,,,
Dendrocopos_major,0,12,1,,13.44,19.513932,15000
Dendrocopos_medius,0,,0,,,,
Dendrocopos_minor,0,3,0,,,,
Dendrocopos_syriacus,0,2,0,,,,

Dryocopus_martius,0,,0,,38.71,17.12696,1070
Egretta_garzetta,0,7,0,,24.5,10.944632,81
Elanus_caeruleus,0,,0,,,,
Emberiza_aureola,0,,0,,,,
Emberiza_bruniceps,0,,0,,,,
Emberiza_buchanani,0,,0,,,,
Emberiza_caesia,0,,0,,,,
Emberiza_cia,0,,0,,9.43,10.609419,2700
Emberiza_cineracea,0,,0,,,,
Emberiza_cirlus,0,,0,,,,
Emberiza_citrinella,1,1,0,0.25,9.99,15.714281,24500
Emberiza_hortulana,0,2,0,0,,,
Emberiza_leucocephalos,0,,0,,,,
Emberiza_melanocephala,0,,0,,,,
Emberiza_pallasi,0,,0,,,,
Emberiza_pusilla,0,,0,,,,
Emberiza_rustica,0,,0,,,,
Emberiza_schoeniclus,0,3,0,0,9.81,18.653395,6800
Emberiza_striolata,0,,0,,,,
Eremalauda_dunni,0,,0,,,,
Eremophila_alpestris,0,,0,,,,
Eremophila_bilopha,0,,0,,,,
Eremopsaltria_mongolicus,0,,0,,,,
Eremopterix_nigriceps,0,,0,,,,
Erithacus_rubecula,1,8,1,0,5.13,22.011569,63000
Erythropygia_galactotes,0,,0,,,,
Eudromias_morinellus,0,2,0,,,,
Falco_biarmicus,0,2,0,,,,
Falco_cherrug,0,,0,,,,
Falco_columbarius,0,10,0,,,,
Falco_eleonorae,0,2,0,,,,
Falco_naumanni,0,,1,,,,
Falco_peregrinus,0,14,1,,,,
Falco_rusticolus,0,,0,,,,
Falco_subbuteo,0,6,0,,,,
Falco_tinnunculus,0,24,1,,30.94,22.234286,415
Falco_vespertinus,0,2,0,,,,
Ficedula_albicollis,0,2,0,,,,
Ficedula_hypoleuca,0,4,1,,,,
Ficedula_parva,0,2,0,,,,
Ficedula_semitorquata,0,,0,,,,
Francolinus_francolinus,0,,0,,,,
Fratercula_arctica,0,,0,,,,
Fringilla_coelebs,0,8,1,0.17,7.14,22.446774,185000
Fringilla_montifringilla,0,3,0,0,,,
Fringilla_teydea,0,,0,,,,
Fulica_atra,1,4,0,,18.57,20.439356,1800
Fulica_cristata,0,,0,,,,
Fulmarus_glacialis,0,,0,,,,
Galerida_cristata,0,,1,,14.33,14.667779,5600
Galerida_theklae,0,,0,,7.18,5.077273,1800
Gallinago_gallinago,0,2,0,,25.83,22.446774,1415
Gallinago_media,0,,0,,,,
Gallinago_stenura,0,,0,,,,
Gallinula_chloropus,1,14,1,,9.88,82.850373,1300
Garrulus_glandarius,0,11,1,,11.76,17.963092,9500
Gavia_arctica,0,2,0,,,,
Gavia_immer,0,,0,,,,

Gavia_stellata,0,2,0,,,,
Geronticus_eremita,0,,0,,,,
Glareola_nordmanni,0,,0,,,,
Glareola_pratincola,0,2,0,,,,
Glaucidium_passerinum,0,,0,,,,
Grus_grus,0,,0,,,,
Grus_virgo,0,,0,,,,
Gypaetus_barbatus,0,,0,,,,
Gyps_fulvus,0,2,0,,,,
Haematopus_ostralegus,0,3,1,,,,
Haliaeetus_albicilla,1,,0,,,,
Hieraaetus_pennatus,0,,0,,,,
Himantopus_himantopus,0,2,0,,,,
Hippolais_caligata,0,,0,,,,
Hippolais_icterina,0,,1,,7.52,14.133265,5300
Hippolais_languida,0,,0,,,,
Hippolais_olivetorum,0,,0,,,,
Hippolais_pallida,0,,0,,,,
Hippolais_polyglotta,0,2,0,,,,
Hirundo_daurica,0,,0,,,,
Hirundo_rupestris,0,,0,,,,
Hirundo_rustica,1,15,1,,10.16,18.42347,26000
Histrionicus_histrionicus,0,,0,,,,
Hydrobates_pelagicus,0,,0,,,,
Hypocolius_ampelinus,0,,0,,,,
Irania_gutturalis,0,,0,,,,
Ixobrychus_minutus,0,,0,,,,
Jynx_torquilla,0,2,0,,,,
Lagopus_lagopus,0,,0,,,,
Lagopus_muta,0,,0,,,,
Lanius_collurio,0,5,0,,7.27,9.350266,9650
Lanius_excubitor,0,10,0,,21.16,20.096647,325
Lanius_isabellinus,0,,0,,,,
Lanius_minor,0,3,0,,,,
Lanius_nubicus,0,,0,,,,
Lanius_senator,0,4,0,,10.99,11.686314,840
Larus_argentatus,0,17,1,,40.15,12.397288,1525
Larus_armenicus,0,,0,,,,
Larus_audouinii,0,,0,,,,
Larus_canus,0,9,0,,59.94,12.299926,1045
Larus_fuscus,0,2,0,,37,18.673625,325
Larus_genei,0,,0,,,,
Larus_glaucoides,0,2,0,,,,
Larus_hyperboreus,0,,0,,,,
Larus_ichthyaetus,0,,0,,,,
Larus_marinus,0,7,0,,68,24.007037,145
Larus_melanocephalus,0,3,0,,,,
Larus_minutus,0,3,0,,,,
Larus_ridibundus,1,26,0,,41.2,19.694179,1850
Limicola_falcinellus,0,,0,,,,
Limosa_lapponica,0,,0,,,,
Limosa_limosa,0,,0,,,,
Locustella_fluviatilis,0,,0,,,,
Locustella_lanceolata,0,,0,,,,
Locustella_luscinioides,0,,0,,,,
Locustella_naevia,0,,0,,15.03,15.493382,1520
Loxia_curvirostra,1,9,0,,4.74,17.613905,9400
Loxia_leucoptera,1,,0,,,,

Loxia_pytyopsittacus,0,,0,,,,
Loxia_scotica,0,,0,,,,
Lullula_arborea,0,,0,0,12.14,14.535017,2300
Luscinia_calliope,0,,0,,,,
Luscinia_luscinia,0,,0,,15.89,10.193761,5300
Luscinia_megarhynchos,0,2,0,0,8.16,11.843585,8100
Luscinia_svecica,0,3,0,,,,
Lymnocryptes_minimus,0,,0,,,,
Marmaronetta_angustirostris,0,,0,,,,
Melanitta_fusca,0,,0,,,,
Melanitta_nigra,0,,0,,,,
Melanocorypha_bimaculata,0,,0,,,,
Melanocorypha_calandra,0,,0,,,,
Melanocorypha_leucoptera,0,,0,,,,
Melanocorypha_yeltoniensis,0,,0,,,,
Mergellus_albellus,0,2,0,,,,
Mergus_merganser,0,2,0,,,,
Mergus_serrator,0,,0,,,,
Merops_apiaster,0,3,0,,37.02,13.583929,740
Miliaria_calandra,0,,0,,9.13,15.792159,14950
Milvus_migrans,0,6,0,,37.91,20.337192,82
Milvus_milvus,0,3,0,,,,
Monticola_saxatilis,0,,0,,16.25,13.40591,210
Monticola_solitarius,1,2,0,,,,
Montifringilla_nivalis,0,,0,,,,
Morus_bassanus,0,4,0,,,,
Motacilla_alba,0,10,1,,11.32,22.41973,19500
Motacilla_cinerea,0,2,0,,7.07,22.129083,1170
Motacilla_citreola,0,,0,,,,
Motacilla_flava,0,2,0,,11.24,20.154247,10950
Muscicapa_striata,0,9,1,,8.5,18.450714,18000
Nectarinia_osea,0,,1,,,,
Neophron_percnopterus,0,2,0,,,,
Netta_rufina,0,2,0,,,,
Nucifraga_caryocatactes,0,4,0,,,,
Numenius_arquata,0,4,0,,62.75,13.184494,290
Numenius_phaeopus,0,,0,,,,
Nycticorax_nycticorax,0,,0,,,,
Oceanodroma_castro,0,,0,,,,
Oceanodroma_leucorhoa,0,2,0,,,,
Oenanthe_albonigra,0,,0,,,,
Oenanthe_deserti,0,2,0,,,,
Oenanthe_finschii,0,,0,,,,
Oenanthe_hispanica,0,,0,,,,
Oenanthe_isabellina,0,,0,,,,
Oenanthe_leucopyga,0,,0,,,,
Oenanthe_leucura,0,2,0,,30.37,2.321398,10
Oenanthe_lugens,0,,0,,,,
Oenanthe_moesta,0,,0,,,,
Oenanthe_monacha,0,,0,,,,
Oenanthe_oenanthe,0,4,0,,15.8,22.41973,8800
Oenanthe_pleschanka,0,,0,,,,
Oenanthe_xanthoprymna,0,,0,,,,
Oriolus_oriolus,0,4,0,,39.67,15.491271,5250
Otis_tarda,0,3,0,,,,
Otus_scops,0,,1,,,,
Oxyura_leucocephala,0,,0,,,,
Pandion_haliaetus,0,8,0,,,,

Panurus_biarmicus,0,,0,,,,
Parus_ater,1,5,0,,5.55,17.463049,20500
Parus_caeruleus,1,12,1,,5.54,18.836186,32000
Parus_cinctus,0,,0,,,,
Parus_cristatus,0,2,0,,6.32,16.272355,9050
Parus_cyanus,0,,0,,,,
Parus_lugubris,0,2,0,,,,
Parus_major,1,16,1,,5.22,18.706806,68500
Parus_montanus,1,2,0,,,,
Parus_palustris,1,5,0,,5.59,15.958623,4500
Passer_domesticus,1,20,1,0.8,3.83,19.222361,96500
Passer_hispaniolensis,0,,0,,11.94,8.602482,4500
Passer_iagoensis,0,,0,,,,
Passer_moabiticus,0,,0,,,,
Passer_montanus,1,5,1,0.6,5.11,18.340824,37000
Pelagodroma_marina,0,,0,,,,
Pelecanus_crispus,0,,0,,,,
Pelecanus_onocrotalus,0,,0,,,,
Perdix_perdix,0,,0,0.48,24.78,15.3452,2350
Perisoreus_infaustus,1,,0,,,,
Pernis_apivorus,0,2,0,,,,
Petronia_brachydactyla,0,,0,,,,
Petronia_petronia,0,,0,,,,
Petronia_xanthocollis,0,,0,,,,
Phalacrocorax_aristotelis,0,3,0,,,,
Phalacrocorax_carbo,1,8,0,,75.29,22.453518,340
Phalacrocorax_pygmeus,0,,0,,,,
Phalaropus_fulicarius,0,2,0,,,,
Phalaropus_lobatus,0,,0,,,,
Philomachus_pugnax,0,4,0,,,,
Phoenicopterus_ruber,0,,0,,,,
Phoenicurus_erythrogastrus,0,,0,,,,
Phoenicurus_erythronotus,0,,0,,,,
Phoenicurus_moussieri,0,,0,,,,
Phoenicurus_ochruros,0,2,1,,6.95,14.506612,6400
Phoenicurus_phoenicurus,0,2,1,,9.1,18.043482,11400
Phylloscopus_bonelli,0,,0,,4.95,8.596744,2450
Phylloscopus_borealis,0,,0,,,,
Phylloscopus_canariensis,0,,0,,,,
Phylloscopus_collybita,0,9,1,,6.49,20.356151,45445
Phylloscopus_inornatus,0,2,0,,,,
Phylloscopus_nitidus,0,,0,,,,
Phylloscopus_sibilatrix,0,,0,,,,
Phylloscopus_sindianus,0,,0,,,,
Phylloscopus_trochiloides,0,,0,,,,
Phylloscopus_trochilus,0,3,0,,6.28,13.133242,78000
Pica_pica,0,19,1,,14.37,19.41778,13250
Picoides_tridactylus,0,,0,,,,
Picus_canus,0,2,0,,,,
Picus_viridis,0,8,1,,17,15.594026,945
Pinicola_enucleator,1,,0,,,,
Platalea_leucorodia,0,,0,,,,
Plectrophenax_nivalis,0,2,0,,,,
Plegadis_falcinellus,0,,0,,,,
Pluvialis_apricaria,0,,0,,,,
Pluvialis_squatarola,0,2,0,,51,1.544686,7
Podiceps_auritus,0,2,0,,,,
Podiceps_cristatus,0,3,0,,,,

Podiceps_grisegena,0,2,0,,,,
Podiceps_nigricollis,0,2,0,,,,
Polysticta_stelleri,0,,0,,,,
Porphyrio_porphyrio,0,,0,,,,
Porzana_parva,0,,0,,,,
Porzana_porzana,0,2,0,,,,
Porzana_pusilla,0,2,0,,,,
Prinia_gracilis,0,,0,,,,
Prunella_atrogularis,0,,0,,,,
Prunella_collaris,0,,0,,,,
Prunella_modularis,0,6,1,1,5.08,17.187814,19000
Prunella_montanella,0,,0,,,,
Prunella_ocularis,0,,0,,,,
Pterocles_alchata,0,,0,,,,
Pterocles_orientalis,0,,0,,,,
Pterodroma_feae,0,,0,,,,
Pterodroma_madeira,0,,0,,,,
Puffinus_assimilis,0,,0,,,,
Puffinus_mauretanicus,0,,0,,,,
Puffinus_puffinus,0,4,0,,,,
Puffinus_yelkouan,0,,0,,,,
Pycnonotus_leucogenys,0,,0,,,,
Pyrrhocorax_graculus,0,2,0,,,,
Pyrrhocorax_pyrrhocorax,0,7,0,,,,
Pyrrhula_murina,0,,0,,,,
Pyrrhula_pyrrhula,0,6,0,0,6.01,18.402914,10650
Rallus_aquaticus,0,10,0,,,,
Recurvirostra_avosetta,0,2,0,,,,
Regulus_ignicapilla,0,2,0,,5.25,9.433512,5000
Regulus_madeirensis,0,,0,,,,
Regulus_regulus,0,6,0,,4.49,19.971091,27000
Remiz_pendulinus,0,2,0,,6.17,13.988146,315
Rhamphocoris_clotbey,0,,0,,,,
Rhodopechys_obsoletus,0,,0,,,,
Rhodopechys_sanguineus,0,,0,,,,
Riparia_riparia,0,7,0,,23.1,18.437661,7450
Rissa_tridactyla,1,5,0,,,,
Saxicola_dacotiae,0,,0,,,,
Saxicola_rubetra,0,2,0,,16.65,16.611916,7700
Saxicola_torquatus,0,6,0,,9.27,17.34553,3300
Scolopax_rusticola,0,3,0,,,,
Scotocerca_inquieta,0,,0,,,,
Serinus_canaria,0,,0,,,,
Serinus_citrinelloides,0,,0,,,,
Serinus_pusillus,0,,0,,,,
Serinus_serinus,0,,1,,5.84,12.823702,14150
Serinus_syriacus,0,,0,,,,
Sitta_europaea,1,4,0,,6.59,16.995436,13250
Sitta_krueperi,0,,0,,,,
Sitta_ledanti,0,,0,,,,
Sitta_neumayer,0,,0,,,,
Sitta_tephronota,0,,0,,,,
Sitta_whiteheadi,0,,0,,,,
Somateria_mollissima,0,2,0,,,,
Somateria_spectabilis,0,,0,,,,
Stercorarius_longicaudus,0,3,0,,,,
Stercorarius_parasiticus,0,,0,,,,
Stercorarius_pomarinus,0,4,0,,,,

Sterna_albifrons,0,2,0,,,,
Sterna_caspia,0,,0,,,,
Sterna_dougallii,0,,0,,,,
Sterna_hirundo,0,9,0,,,,
Sterna_nilotica,0,2,0,,,,
Sterna_paradisaea,0,3,0,,,,
Sterna_sandvicensis,0,4,0,,,,
Stigmatopelia_senegalensis,0,,0,,,,
Streptopelia_decaocto,1,4,1,1,5.38,18.103545,7850
Streptopelia_turtur,0,2,0,0,,,
Strix_aluco,0,,1,,,,
Strix_nebulosa,0,,0,,,,
Strix_uralensis,0,,0,,,,
Sturnus_roseus,0,,0,,,,
Sturnus_unicolor,0,,1,,14.3,2.134573,2600
Sturnus_vulgaris,1,15,1,0.64,9.75,21.868439,39500
Surnia_ulula,0,,0,,,,
Sylvia_atricapilla,0,11,1,0,5.84,22.047377,37000
Sylvia_borin,0,,0,,6.6,16.642795,24000
Sylvia_cantillans,0,,0,,5.69,4.154768,2300
Sylvia_communis,0,4,0,0,8.15,18.087278,19500
Sylvia_conspicillata,0,,0,,7.27,6.41215,310
Sylvia_curruca,0,2,1,,5.28,16.741079,6300
Sylvia_deserticola,0,,0,,,,
Sylvia_hortensis,0,,0,,,,
Sylvia_leucomelaena,0,,0,,,,
Sylvia_melanocephala,0,,0,,6.64,5.640381,5600
Sylvia_melanothorax,0,,0,,,,
Sylvia_mystacea,0,,0,,,,
Sylvia_nana,0,,0,,,,
Sylvia_nisoria,0,2,0,,,,
Sylvia_rueppelli,0,,0,,,,
Sylvia_sarda,0,,0,,,,
Sylvia_undata,0,,0,,10.16,4.031074,2800
Tachybaptus_ruficollis,0,6,0,,,,
Tachymarptis_melba,0,2,1,,,,
Tadorna_ferruginea,0,,0,,,,
Tadorna_tadorna,0,2,0,,36.3,17.039171,54
Tarsiger_cyanurus,0,,0,,,,
Tetrao_mlokosiewiczi,0,,0,,,,
Tetrao_tetrix,0,,0,0,,,
Tetrao_urogallus,0,,0,,,,
Tetraogallus_caspius,0,,0,,,,
Tetraogallus_caucasicus,0,,0,,,,
Tetrax_tetrax,0,,0,,,,
Tichodroma_muraria,0,,0,,,,
Tringa_erythropus,0,3,0,,,,
Tringa_glareola,0,,0,,,,
Tringa_nebularia,0,6,0,,30,8.960859,118
Tringa_ochropus,0,,0,,,,
Tringa_stagnatilis,0,,0,,,,
Tringa_totanus,0,4,0,,29.71,22.229241,445
Troglodytes_troglodytes,0,3,1,,5.64,21.608446,31500
Turdoides_altirostris,0,,0,,,,
Turdoides_squamiceps,0,,0,,,,
Turdus_iliacus,0,5,1,,12.75,11.446024,18500
Turdus_merula,1,31,1,0.57,7.11,22.41973,61000
Turdus_philomelos,0,13,1,0.38,7.73,17.678168,28000

Turdus_pilaris,0,6,1,,14.21,11.244834,19000
Turdus_ruficollis,0,,0,,,,
Turdus_torquatus,0,,0,,,,
Turdus_viscivorus,0,6,0,,19.33,17.984922,5200
Tyto_alba,0,,1,,,,
Upupa_epops,0,,0,,19.96,16.14043,1295
Uria_aalge,0,4,0,,,,
Uria_lomvia,0,,0,,,,
Vanellus_vanellus,0,4,0,0,34.69,17.776382,2250
Xenus_cinereus,0,,0,,,,
